# *Artocarpus tonkinensis* Protects Mice Against Collagen-Induced Arthritis and Decreases Th17 Cell Function

**DOI:** 10.3389/fphar.2019.00503

**Published:** 2019-05-31

**Authors:** Sabrina Adorisio, Alessandra Fierabracci, Isabella Muscari, Anna Marina Liberati, Mario Calvitti, Lina Cossignani, Francesca Blasi, Tran Duc Quan, Nguyen Thanh Tam, Tran Van Sung, Carlo Riccardi, Trinh Thi Thuy, Domenico V. Delfino

**Affiliations:** ^1^Department of Medicine, Foligno Nursing School, University of Perugia, Foligno, Italy; ^2^Infectivology and Clinical Trials Research Area, Bambino Gesù Children’s Hospital, IRCCS, Rome, Italy; ^3^Section of Onco-hematology, Department of Medicine, Santa Maria Hospital, University of Perugia, Terni, Italy; ^4^Department of Experimental Medicine, University of Perugia, Perugia, Italy; ^5^Department of Pharmaceutical Sciences, Section of Food Science and Nutrition, University of Perugia, Perugia, Italy; ^6^Institute of Chemistry, Vietnam Academy of Science and Technology, Hanoi, Vietnam; ^7^Graduate University of Science and Technology, Vietnam Academy of Science and Technology, Hanoi, Vietnam; ^8^Section of Pharmacology, Department of Medicine, University of Perugia, Perugia, Italy

**Keywords:** medicinal plant, interleukin-17, Vietnam traditional medicine, autoimmunity, Hmong ethnic minority, rheumatoid arthritis

## Abstract

*Artocarpus tonkinensis* (Moraceae) is a tree that grows in north Vietnam whose leaf decoction is used as a traditional remedy by the Hmong ethnic group to treat arthritis and backache. Our study evaluated the decoction’s efficacy and mechanism of action in DBA/1J mice with collagen-induced arthritis (CIA). Mice treated with the decoction (At) either from the first collagen immunization or after CIA development experienced significantly less joint edema and inflammatory infiltration, whereas CIA-induced cartilage damage could only be prevented by early At treatment. Autoimmune gene expression profiles showed that Th17 cell-associated chemokine CCL20 and cytokines IL-6, IL-17, and IL-22 were strongly downregulated by At. Reduced expression of *IL-2*, *IL-17*, *IL-22*, and *FasL* in lymph node cells from At-treated mice was further confirmed by real-time PCR. The decoction also inhibited polarization of Th17 cells from CD4^+^ splenic T cells according to levels of IL-17 and RORC, a Th17 cell-specific transcription factor. Chromatographic analysis identified At’s major component as maesopsin-β-D-glucoside, which could inhibit *in vitro* differentiation of Th17 cells. The decoction significantly alleviated the signs and symptoms of CIA and inhibited the development and function of Th17 cells, highlighting its potent anti-inflammatory activity.

## Introduction

Rheumatoid arthritis (RA) is an autoimmune disease characterized by bone and joint destruction (Kuwabara et al., 2017) directly related to changes in joint structure. Bone destruction occurs due to osteoclasts that differentiate from monocytes under the influence of receptor activator of nuclear factor kappa-B ligand (RANKL) (Horwood et al., 1999; Kong et al., 1999). Helper T cells infiltrating the joints play a pivotal role in this process, and Th17 cells specifically have been found in the synovial tissue of RA patients (Kotake et al., 1999). Interleukin (IL)-17 induces RANKL expression and stimulates osteoclast development and is critical for RA pathogenesis in humans and in experimental animal models with collagen-induced arthritis (CIA) (Nakae et al., 2003; Sarkar et al., 2007). After the discovery of Th17 cells in Sato et al. (2006), the RANKL-dependent induction mechanism of osteoclasts via Th17 cells in RA became clear. Both RA and CIA are also characterized by chronic joint inflammation mediated by IL-22 produced by Th17 cells, which stimulates synovial fibroblasts to induce cell proliferation and secretion of other inflammatory cytokines (Colin et al., 2010), and the initiation of osteoclastogenesis (Geboes et al., 2009). Moreover, IL-6 produced by fibroblasts responding to Th17 cell-derived IL-17 amplifies inflammation (Ogura et al., 2008) and stimulates synovial tissue in an autocrine manner; thus, IL-17 maintains the inflammatory cycle via activity of downstream cytokines.

Ethnopharmacology research of traditional medicine disciplines is a potentially valuable strategy for discovering new drugs for RA treatment. In traditional Chinese medicine (TCM), for example, RA is considered to arise from an imbalance between “wind (feng),” “damp (shi),” and “cold (han),” and different TCM formulas, validated by modern biotechnological tools, are available that act by dissipating wind and damp and rebalancing the blood circulation (Lu et al., 2015).

Traditional Chinese medicine had a major influence on Vietnamese traditional medicine (VTM). VTM includes theories, beliefs, and practices of medicine derived from Vietnamese culture, which are also integrated with Western medicine concepts. VTM is based mainly on acupuncture; herbal, animal, and mineral medicines; moxibustion; psycho-physical practices (e.g., QiGong); Tai Chi; and massages (Dung and Bodeker, 2001; Adorisio et al., 2016). Chewing betel (or *trau cau*) derived from areca nuts, lime paste, and *Artocarpus tonkinensis* bark is among the VTM practices resulting from medical exchanges with China (Liu and Dai, 2012).

*Artocarpus tonkinensis* (A.Chev. ex Gagnep) is an ornamental tree from the Moraceae family that grows in northern Vietnam and is used in VTM by the Hmong ethnic minority to treat arthritis and backache. The medicinal use of its dried leaf decoction was discovered by Pham Chuk Lam and later studied for its immunosuppressive activity in arthritis, myasthenia gravis, and skin transplants. Different active compounds have been isolated from *A. tonkinensis* leaves (Thuy et al., 2017), such as the anti-arthritic *n*-butanol extract containing the immunosuppressive auronol glycosides maesopsin 4-*O*-glucoside and alphitonin-4-*O*-glucoside (Thuy et al., 2004). Intraperitoneal injection of its ethyl acetate extract decreased both arthritis incidence and severity and delayed disease onset in rats with CIA. The extract also inhibited mitogen-induced T cell proliferation and stimulated apoptosis of activated lymph node-derived lymphocytes (Ngoc et al., 2005).

In addition, four individual active components isolated from *A. tonkinensis* have anti-inflammatory effects that correlate with its inhibition of mitogen-induced T cell proliferation. These extracts inhibited production of inflammatory cytokines, such as tumor necrosis factor-α and interferon-γ, in mitogen-stimulated T cells. Suppression of T cell proliferation and cytokine production by *A. tonkinensis* flavonoids may reduce disease severity after experimentally induced arthritis (Dang et al., 2009). In anti-cancer trials, maesopsin 4-*O*-β-D-glucoside (TAT-2) showed antiproliferative effects on acute myeloid leukemia cells and modulated expression of 19 cancer-related genes encoding proteins such as heme oxygenase-1, sulfiredoxin 1 homolog, and breast carcinoma amplified sequence 3 (Pozzesi et al., 2011), and exhibited *in vivo* anti-cancer effects (Thuy et al., 2016).

Our current study further explored the *in vivo* and *in vitro* immunoregulatory activity of *A. tonkinensis* extracts. We analyzed differential gene expression and immune system involvement in an established CIA mouse model and examined the influence of *A. tonkinensis* decoction on lymphocyte subpopulation involvement and cytokine production during lymphocyte activation and Th17 cell differentiation. Our results suggest that *A. tonkinensis* inhibits the maturation and/or function of Th17 cells involved in the development of arthritis.

## Materials and Methods

### Eliciting CIA in Mice

DBA/1J female mice, 8–12 weeks old, were supplied by Biogem SCARL (Ariano Irpino, Italy). C57BL/6 female mice and additional female DBA/1J mice were purchased from Charles River (Wilmington, MA, United States) and housed in the animal facility of the University of Perugia. CIA was elicited by intradermal immunization with 50 μL/mouse collagen type II (Sigma, C9301-5MG, Lot#016M4158V; St. Louis, MO, United States) emulsified in complete Freund’s adjuvant (Sigma, F5881-10ML, Lot#SLBQ1106V) on day 1 and in incomplete Freund’s adjuvant (Sigma, F5506-10ML, Lot#SLBL9742V) on day 31. Arthritis was induced in 15 mice (3 groups of 5 mice each), while 3 mice composed the untreated negative control group. An additional 12 mice (4 mice/group) were immunized and utilized for gene expression profiling. Complete details of CIA mouse generation are provided in the Supplementary Material. Details of the CIA experimental procedure, with the arthritis score for each mouse, are given in Supplementary Material. Animal experiments were performed after approval by the ethical committee of the University of Perugia.

### *A. tonkinensis* Extracts

Leaves of *A. tonkinensis* were harvested in Hanoi, Vietnam in October 2015, and the voucher species (No. AT-2015) was deposited in the Institute of Chemistry, Vietnam Academy of Science and Technology. Briefly, freshly harvested leaves were air-dried for 2 days and oven-dried at 40–50°C in a forced-air oven. To obtain the decoction, 5 g dried *A. tonkinensis* leaves were boiled 3 times in a total volume of 100 mL (5 g/100 mL final concentration) distilled water. The decoction was then given to mice *ad libitum* instead of water (At-treated mice). For *in vitro* studies, the decoction was prepared by boiling dried leaves in RPMI-1640 medium and filtering the total extract with a 0.22-μm syringe filter (5 g/100 mL final concentration). The TAT-2 compound was isolated as previously described (Pozzesi et al., 2011).

### Histological Assessment

One knee joint per mouse was removed, fixed in 10% (v/v) formalin for 24 h, decalcified in 5% (v/v) trichloroacetic acid for 7 days, dehydrated, embedded in paraffin, sectioned into 3- to 4-μm-thick sections, and stained with hematoxylin and eosin.

### Autoimmune Gene Expression Profiling

The Qiagen Inflammatory Response and Autoimmunity RT^2^ Profiler PCR Array was utilized according to the manufacturer’s protocol (Valencia, CA, United States). Briefly, the conversion of experimental mRNA to first-strand cDNA was accomplished using the RT^2^ First Strand Kit. Next, cDNA was added to the appropriate RT^2^ SYBR Green Mastermix, which was aliquoted into wells of the RT^2^ Profiler PCR Array. Relative gene expression was determined using the ΔΔCt method.

### Real-Time (RT)-PCR

RNA was isolated using the Qiagen RNeasy Plus Micro kit, and conversion of total RNA to cDNA was performed with the QuantiTect Reverse Transcription kit (Qiagen). RT-PCR was performed with the ABI-7300 Real-Time Cycler (Applied Biosystems, Foster City, CA, United States), and amplification was achieved using the TaqMan Assay (Mm00439618 for *IL-17a*, Mm04209823 for *IL-22*, Mm01261022 for *RORC*, Mm00438864 for *FasL*, and Mm00434256 for *IL-2* (Thermo Fisher Scientific, Waltham, MA, United States). The ΔΔCt method was used to determine the expression levels of *IL-17a*, *IL-22, IL-2*, *FasL*, and *RORC*).

### Isolation of CD4^+^T Cells and Th17 Polarization

CD4^+^ T cells were isolated (>95% purity, see Supplementary Material) from single-cell suspensions of mouse spleens using the Naive CD4^+^ T Cell Isolation kit (Miltenyi Biotec, Bergisch Gladbach, Germany). After isolation, cells were seeded on 24-well plates at a concentration of 1 × 10^6^ cells/well and maintained in RPMI 1640 medium with 10% fetal bovine serum, 100 U/mL penicillin, and 100 μg/mL streptomycin at 37°C and with 5% CO_2_ for 6 days. CD4^+^ T cells were activated with anti-mouse CD3 and anti-mouse CD28 (eBioscience, Thermo Fisher Scientific). For Th17 polarization, we used anti-mouse IL-12, IL-23, p40 (eBioscence), anti-mouse IL-4, (eBioscence), recombinant murine IL-6 (Peprotech, Rocky Hill, NJ, United States), and recombinant human TGF-β1 (Peprotech).

### HPLC-DAD Profiling of *A. tonkinensis* Leaf Decoction

A Spectra System HPLC system (Thermo Separation, San Jose, CA, United States), fitted with a quaternary pump module (P4000), an online degasser, and a diode array detector (DAD) SpectroSystem UV 6000lp (Thermo Separation.) was used. Analytes were separated using a reversed-phase Agilent Zorbax ODS column (5-μm particle size, 3.0 × 150 mm i.d.; Agilent Technologies, Milan, Italy), coupled to a 20 × 4.6 mm C18 guard column (Blasi et al., 2016). Gradient elution with a flow rate of 1 mL/min was used. The mobile phase consisted of the following: (A) water containing 0.1% formic acid and (B) acetonitrile containing 0.1% formic acid. The initial mobile phase composition was 95% A. The percentage of B was linearly increased to 20% at 30 min and to 55% at 50 min. Finally, the percentage of B was reduced to 5% and the column re-equilibrated to the initial conditions for 7 min. The injection volume was 20 μL. Detection was performed online using DAD in the range from 200 to 700 nm. Chromatograms were acquired and data were handled using Xcalibur software version 1.2 (Finnigan Corporation 1998–2000, San Jose, CA, United States). A standard solution containing catechin, kaempferol, kaempferol-3-*O*-glucoside, quercetin, quercetin-3-β-D-glucoside (all obtained from Extrasynthese, Genay, France), and TAT-2 (isolated by us) was used to identify and quantify the analytes. Calibration curves were generated using three injections at different concentrations ranging from 1.5 μg/mL to 120.0 μg/mL. TAT-6 was identified by comparing the UV-Vis spectrum with data from the literature (Dang et al., 2009) and quantified using the calibration curve for TAT-2.

### Statistical Analysis

Statistical significance was determined using the Mann-Whitney *U*-test or Student’s *t*-test as specified in the figure legends. Differences were considered statistically significant according to the following criteria: ^∗^*p* < 0.05; ^∗∗^*p* < 0.01; ^∗∗∗^*p* < 0.001.

## Results

### Quality Monitoring of At Water Extract

**FIGURE 1:**
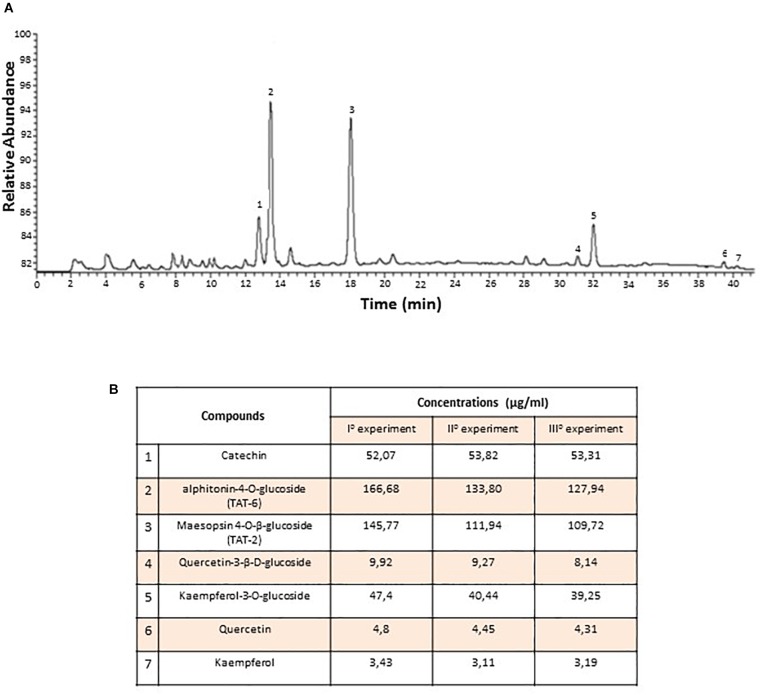
Chemical composition of *Artocarpus tonkinensis* water extract. **(A)** HPLC-DAD profile of phenols from *A. tonkinensis* leaf decoction. **(B)** Identified compounds: (1) catechin, (2) aphitonin-4-*O*-glucoside (TAT-6), (3) maesopsin-4-*O*-β-glucoside (TAT-2), (4) quercetin-3-β-D-glucoside, (5) kaempferol-3-*O*-glucoside, (6) quercetin, and (7) kaempferol.

To determine the chemical composition of the At water extract (decoction) both qualitatively and quantitatively, three independent HPLC experiments were performed. Seven compounds were identified, and the quantities of each compound were consistent among the three experiments (Figure 1A). The most abundant compounds were TAT-2 and TAT-6, with no significant difference in quantity between the two compounds (mean ± standard deviation, 1225.0 ± 16.5 μg/mL and 142.8 ± 17.0 μg/mL, respectively, *p* = 0.29) (Figure 1B). Thus, quality monitoring showed that At water extract composition was quite constant among experiments.

### Effect of At on CIA Progression

**FIGURE 2:**
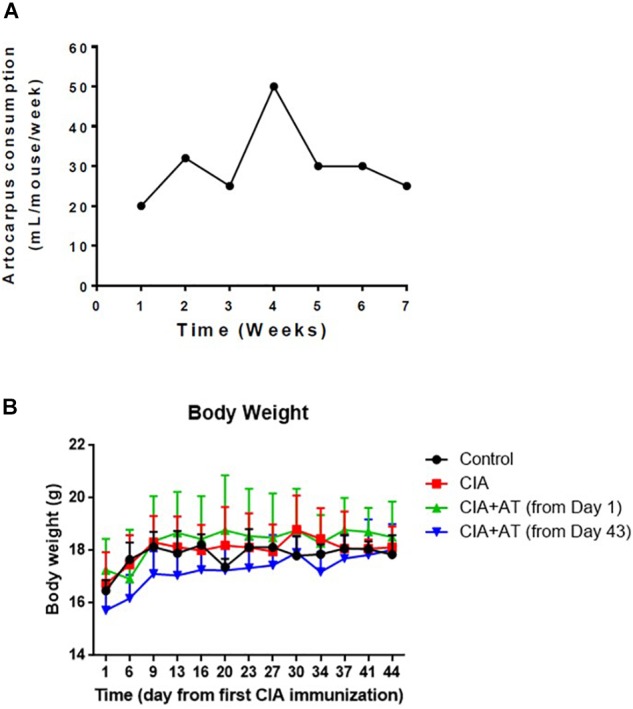
*Artocarpus tonkinensis* decoction consumption and body weight. **(A)** Amount of *A. tonkinensis* decoction (mL/mouse, *y*-axis) consumed by mice during the course of experiment (weeks, *x*-axis). **(B)** Body weight (g) of mice in groups drinking water (Control), immunized and drinking water (CIA), immunized and drinking At decoction from day 1 [CIA + At (from day 1)], and immunized and drinking At decoction from day 43 [CIA + At (from day 43)].

Mice were classified into the following groups: (1) not immunized or treated with At (Control) (*n* = 3); (2) immunized to induce CIA but not treated with At (CIA) (*n* = 5); (3) immunized and treated with At from the first day of immunization [CIA + At (from day 1)](*n* = 5); and (4) immunized and treated with At after development of CIA [CIA + At (from day 43)](*n* = 5). Figure 2A shows that At consumption was generally consistent, with a peak at week 4 (the time of the second immunization): the mean decoction (50 g/L) consumption was 4.4 mL/day/mouse. Consumption of At decoction by CIA mice was higher than that of water by CIA mice, as measured in our sister experiment (see Supplementary Material). The animals’ body weight increased 8–10% by the end of the study, with no significant differences among the four groups (Figure 2B).

**FIGURE 3:**
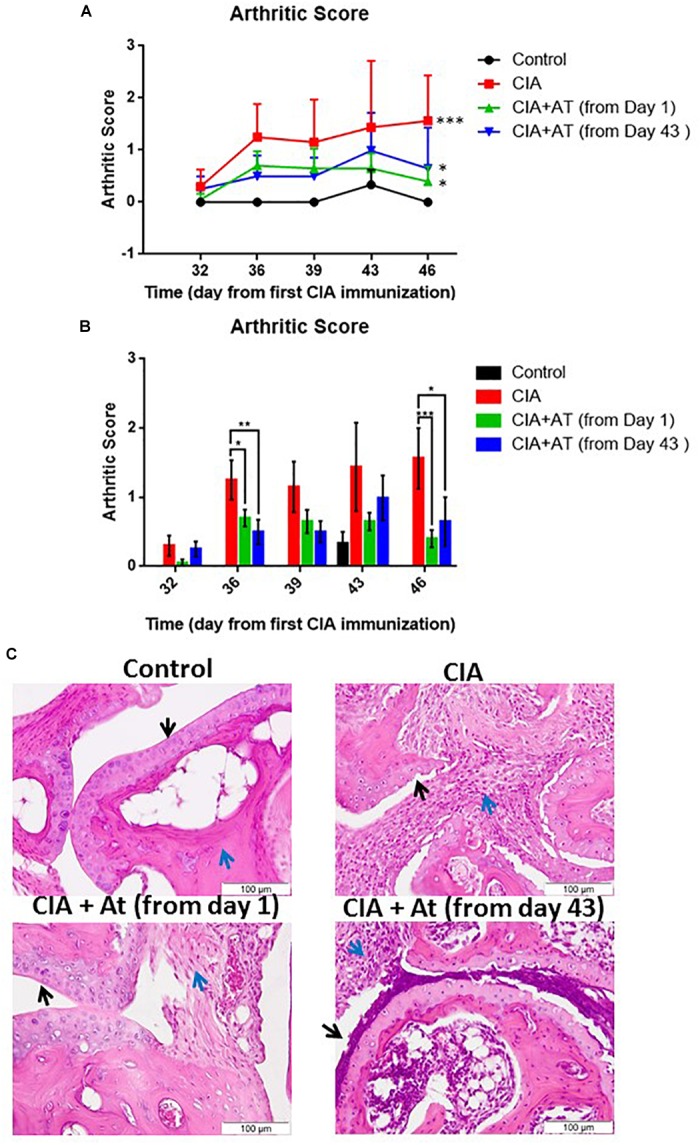
Effect of *A. tonkinensis* decoction on joints. **(A)** Arthritis score (*y*-axis, lines) following the first collagen immunization (days, *x*-axis) of mice in groups drinking water (Control), immunized and drinking water (CIA), immunized and drinking At decoction from day 1 [CIA + At (from day 1)], and immunized and drinking At decoction from day 43 [CIA + At (from day 43)]. **(B)** Arthritis scores (*y*-axis, bars) (bars) during days from the first immunization (*x*-axis) of mice in the groups drinking water (Control), immunized and drinking water (CIA), immunized and drinking At decoction from day 1 [CIA + At (from day 1)], and immunized and drinking At decoction from day 43 [CIA + At (from day 43)]. **(C)** Histological assessment of joint tissue from a representative mouse in each group. Black and blue arrows indicate cartilage and inflammatory infiltrate, respectively, in the joints of mice in groups drinking water (Control, upper left panel), immunized and drinking water (CIA, upper right panel), immunized and drinking At decoction from day 1 [CIA + At (from day 1), lower left panel], and immunized and drinking At decoction from day 43 [CIA + At (from day 43), lower right panel]. Data are presented as mean values + 1 standard deviation (SD). Statistical analysis was performed with the Mann–Whitney *U*-test: ^∗∗∗^*p* < 0.001; ^∗∗^*p* < 0.01; ^∗^*p* < 0.05.

Arthritis induction was scored following the criteria outlined in Supplementary Table S1. Starting at day 32, signs of arthritis appeared, with scores ranging from 1 to 2. Arthritis development became more evident on day 43, with scores ranging from 1 to 4. We further monitored CIA progression through weekly calculation of the arthritis score and observed a markedly increased arthritis score in the CIA-induced mice compared to controls not attributable to normal ankle growth (Figure 3A). Concomitantly with CIA development, we noted clinical signs of suffering that did not compromise the animals’ overall welfare, likely due to limb pain. As the experimental period continued, CIA mice became less active and more easily manipulated. Figure 3B presents the mean arthritis scores of the experimental mice from CIA onset (day 32). At day 46, scores significantly differed between CIA vs. CIA + At (from day 1) and vs. CIA + At (from day 43) mice (*p* < 0.001 and *p* < 0.05, respectively), with approximately 50% decreased scores in At-treated mice compared to untreated mice. Although arthritis development became evident in CIA mice at day 32, administration of At from either day 1 or day 43 significantly reduced CIA progression as evaluated by joint edema.

Figure 3C illustrates joint histology in control and experimental mice. Cartilage was intact and smooth in healthy mice, and no inflammatory infiltrate was present. Mice with CIA exhibited altered cartilage with many visible niches and dramatic inflammatory infiltrate. When At decoction was administered before CIA development, cartilage was intact and smooth as in the control mice, and only a mild inflammatory infiltration was observed. In mice treated with At after CIA development, cartilage was altered similarly to that of CIA untreated mice, but the inflammatory infiltrate was reduced. Thus, At decoction prevents CIA if given before appearance of clinical signs and limits CIA progression after arthritis development, although At does not reverse CIA-related cartilage damage.

### Effect of At on Expression of Autoimmunity-Related Genes in CIA Mice

**FIGURE 4:**
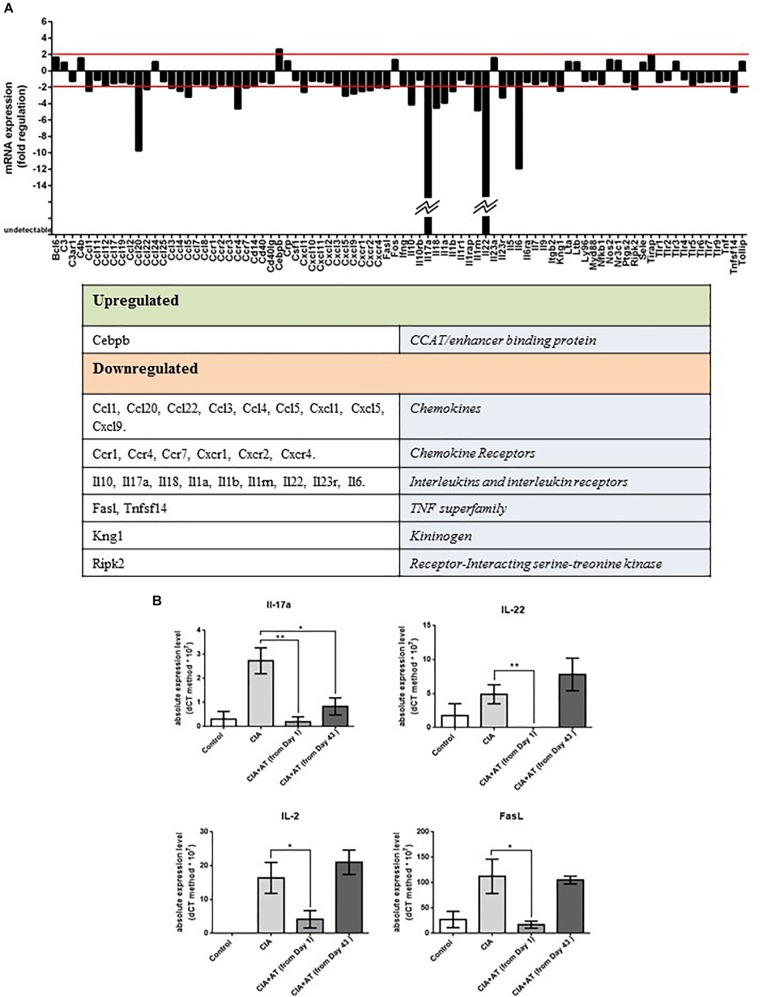
**(A)** Autoimmune gene expression profiles. Genes related to autoimmune responses significantly up- or downregulated by At decoction are shown. Analysis was performed on RNA extracted from 2 CIA mice drinking water and 2 CIA mice drinking At decoction. **(B)** Real-time PCR of targeted autoimmune-related genes from mouse lymph nodes. RNA extracted from lymph nodes of mice in the groups drinking water (Control), immunized and drinking water (CIA), immunized and drinking At decoction from day 1 [CIA + At (from day 1)], and immunized and drinking At decoction from day 43 [CIA + At (from day 43)] was template for real-time PCR of *IL-17* (upper left), *IL-22* (Upper right), *IL-2* (lower left) and *FasL* (lower right). Bars represent values ± 1 SEM from three independent experiments. Statistical analysis was performed with a Student’s *t*-test: ^∗∗^*p* ≤ 0.01; ^∗^*p* ≤ 0.05.

Expression profiles of genes involved in autoimmunity were generated from mRNA extracted from joints of CIA and [CIA + At (from day 1)] mice (Figure 4A). We identified 29 significantly modulated genes, including one upregulated gene encoding CCAAT/enhancer binding protein β, whose expression increased approximately 3-fold with At treatment. The 28 downregulated genes included those encoding 9 chemokines (CCL1, CCL20, CCL22, CCL3, CCL4, CXCL1, CXCL5, and CXCL9), 6 chemokine receptors (CCR1, CCR4, CCR7, CXCR1, CXCR2, and CXCR4), 10 cytokines and cytokine receptors (IL-10, IL-17a, IL-18, IL-1a, IL-1b, IL-1RN, IL-22, IL-23r, IL-6), 3 proteins related to apoptosis (FasL, Ripk2, and TNFSF14), and the kininogen KNG1. Expression of genes encoding IL-17 and IL-22 was not detected in At-treated joints, and *CCL20* and *IL-6* expression decreased more than 10 fold (Figure 4A). These four downregulated genes attracted our attention because they are involved in the differentiation and/or function of Th17 cells. Specifically, the proinflammatory cytokines IL-17 and IL-22 are normally produced by Th17 cells (Pelaia et al., 2012), IL-6 promotes differentiation of Th0 cells to Th17 cells (Pelaia et al., 2012), and CCL20 enhances Th17 cell migration (Kaneko et al., 2018). These gene expression profiles suggest At differentially attenuates Th17 cell-associated immune responses in the joints of CIA mice.

Our gene expression results prompted us to further evaluate expression of Th17 cytokines and T cell activation markers in the lymph nodes of control and experimental mice via RT-PCR. We specifically measured expression of genes encoding IL-2, an activated T cell cytokine (Pozzesi et al., 2007), IL-17, IL-22, and FasL, a marker of T cell activation and apoptosis. Figure 4B shows that expression of *IL-17* was significantly inhibited in all At-treated mice, whereas *IL-2*, *IL-22*, and *FasL* were significantly inhibited in the day 1 At-treated mice only. These expression patterns reflect the partial protective effect of At administered in mice after appearance of CIA signs observed in our histological analysis.

### Effect of At Decoction on Cytokine Expression and Th17 Differentiation in Healthy DBA1J Mice

**FIGURE 5:**
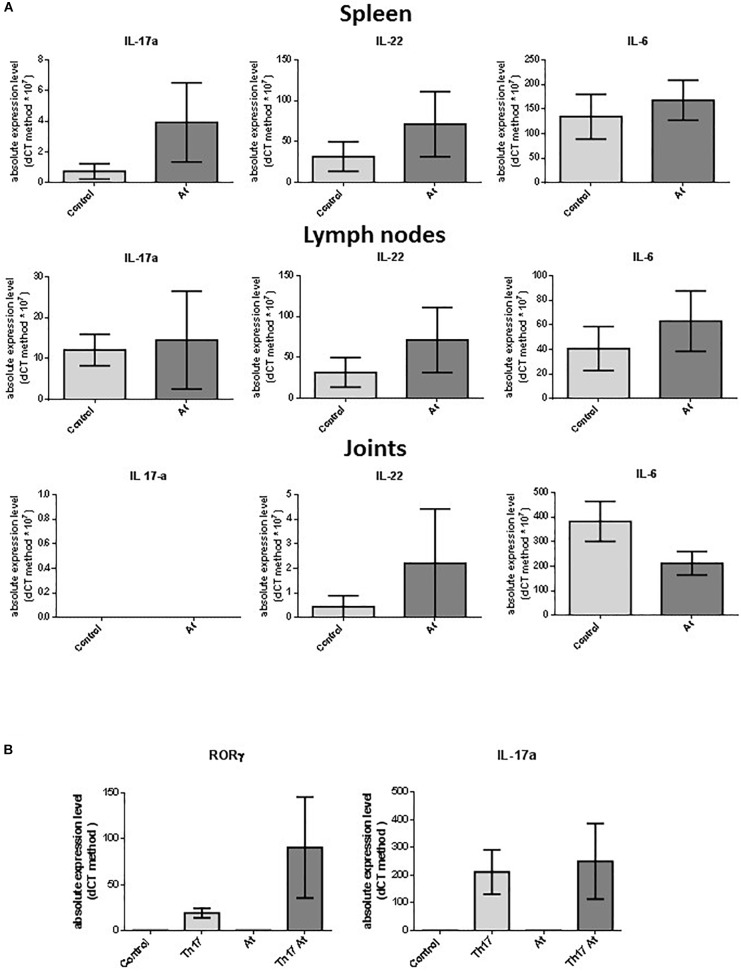
**(A)** Real-time PCR of *Il-17, IL-22*, and *IL-6* in mouse spleen, lymph node, and joint cells. RNA extracted from spleen, lymph node, and joint cells of mice in groups drinking water (light gray bars, Control, *n* = 6) or At decoction (deep gray bars, At, *n* = 5) was used as the template for real-time PCR of *IL-17* (IL-17a), *IL-22* (IL-22), and *IL-6* (IL-6). **(B)** Expression of *RORC* (left) or *IL-17* (right) was measured by real-time PCR. mRNA was extracted from splenic CD4^+^ T lymphocytes obtained from water-drinking or AT decoction-drinking mice, which were either non-polarized (control or At, respectively) or Th17-polarized (Th-17 or Th-17 At, respectively). Bars represent mean values ± 1 SEM from three independent experiments. Statistical analysis was performed with a Student’s *t*-test.

To determine the effects of At ingestion in healthy DBA1J mice (same types of animals used for the CIA *in vivo* experiments), At decoction was administered to these mice at the same concentration used for the aforementioned CIA *in vivo* experiments. After 10 days, the mice were sacrificed, and their spleen, lymph node, and joint cells were harvested. RNA was extracted for further analysis. No macroscopic differences were observed in the mice or their organs between water-treated and At-treated mice. There were no significant differences between the number of spleen and lymph node cells from water- or At-treated mice. As shown in Figure 5A, RT-PCR analyses showed no significant differences in mRNA expression of *IL-6, IL-17*, or *IL-22* in the spleen, lymph node, or joint cells between animal groups. Similarly, no significant differences were detected between water- and At-treated mice in the differentiation of isolated CD4^+^ T cells to Th17 cells, although more variability was observed in the At decoction group (Figure 5B). Thus, 10 days of At decoction was not associated with any significant differences compared to 10 days of water, suggesting that At decoction has no acute toxic or other effects in healthy DBA1J mice.

### Effect of At on *in vitro* Th17 Polarization From Splenic CD4^+^ T Lymphocytes

**FIGURE 6:**
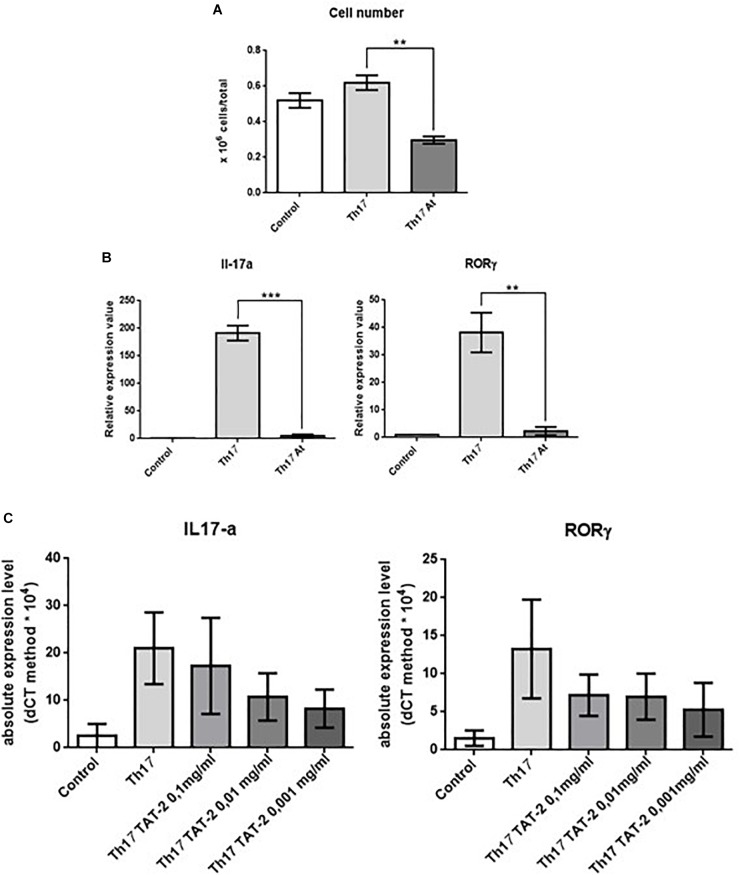
Effect of decoction and TAT-2 on isolated mouse splenic CD4^+^ T lymphocytes. **(A)** Number of untreated mouse splenic CD4^+^ T lymphocytes (Control), Th17-polarized (Th17) cells, and Th17 polarized treated with At (Th17 At). **(B)** Expression of *IL-17* (left) or *RORC* (right) in these groups was measured by real-time PCR. Bars represent values ± 1 SEM from three independent experiments. Statistical analysis was performed with a Student’s *t*-test: ^∗∗∗^*p* ≤ 0.001, ^∗∗^*p* ≤ 0.01. **(C)** Transcription of *IL-17* (left) or *RORC* (right) by real-time PCR in CD4^+^ splenic lymphocytes stimulated with anti-CD3 plus anti-CD28 monoclonal antibodies (Control), polarized to Th17 differentiation (Th17), or polarized and treated with varying concentrations of TAT-2. Bars represent absolute expression levels ± 1 SEM of three independent experiments.

To determine whether inhibition of Th17 cells is a specific property of At water extract, we also analyzed the role of the decoction in the *in vitro* development of Th17 cells. The results of these experiments were not meant to replace the *in vivo* results but to provide further support for the findings of the *in vivo* CIA model suggesting that At decoction attenuates Th17 responses. Splenic CD4^+^ T lymphocytes were isolated from mice and cultured in the following groups: (1) in anti-CD3-adhered plates with anti-CD28 antibodies to stimulate T cell activation; (2) the same as group 1 with the addition of IL-6 and TGF-β to promote differentiation to Th17 cells and the addition of anti-IL-4 and anti-IL-12 monoclonal antibodies (mAbs) to block differentiation to Th1 and Th2 cells, respectively; and (3) same as group 2 with the addition of At. Expression of *IL-17*, *IL-22*, and *RORC*, a Th17 cell-specific transcription factor, in the presence or absence of At was then measured by RT-PCR. As shown in Figure 6A, At significantly decreased the total number of Th17 cells compared to those in untreated cells. Th17 polarization was successful in control cells, but not in At-treated cells, as indicated by the At-mediated inhibition of *IL-17* and *RORC* (Figure 6B). Thus, At inhibited the *in vitro* differentiation of Th17 cells from splenic CD4^+^ T cells.

Since TAT-2 (Pozzesi et al., 2011) is one of the two major components of the At decoction (Figure 1), the effect of TAT-2 on differentiation of Th17 cells from splenic CD4^+^ T lymphocytes was assayed. As shown in Figure 6C, TAT-2 at concentrations of 0.01 and 0.001 mg/mL decreased expression of *IL-17* and *RORC*, and as the primary component of the decoction, likely contributed to the reduced Th17 differentiation in At-treated mice.

## Discussion

The limited options for treatment of RA highlight the need for novel therapeutics. Rather than follow a chemical discovery approach, we functionally tested a VTM-derived herbal remedy for anti-inflammatory activity in CIA mice, an established arthritis experimental model (Brand et al., 2007). Although, recent studies explored the efficacy on RA of the ethyl extract of the leaves of *A. tonkinensis* (Ngoc et al., 2005) and one of its isolated compounds (Dang et al., 2009), we chose to evaluate the At decoction, which has not been heretofore studied, because this is the ethnopharmacological remedy originally used by the Hmong minority in North Vietnam. The main goals were to validate the clinical usefulness of At dedoction for RA and to explore its mechanism of action. To this end, the current study compared the effects of drinking At water extract to the effects of drinking water, an inert (control) fluid. We first monitored the composition of the At decoction and verified that the composition was consistent among different samples. Subsequent experiments in CIA mice showed that ingesting At decoction significantly alleviated signs and symptoms of arthritis, increased the mice’s activity, and reduced their joint edema when given both prior to and after CIA development. However, cartilage damage that occurred prior to At administration could not be reversed under the conditions we tested, although the possibility that more long-term treatment of At could rescue CIA-impaired cartilage should be explored.

The autoimmune gene expression profiles of joints from At-treated mice showed downregulation of 28 genes, 4 of which encode proteins related to development and function of Th17 cells (IL-6, IL-17, IL-22, and CCL20) and indirectly contribute to RA pathogenesis. Specifically, Th17 cells differentiate in the lymphoid organs under stimulation of IL-6 and TGF-β and subsequently produce IL-17 and IL-22. Additionally Th17 cells migrate to the joints due to CCL20’s chemoattractant activity. IL-6 also inhibits differentiation of T regulatory cells (Pelaia et al., 2012) produced in the thymus and peripheral lymph nodes to protect against autoimmunity (Delfino et al., 2011). Thus, the absence of IL-17 and IL-22 and decreased expression of *IL-6* and *CCL20* in At-treated mice strongly suggests an At-mediated mechanism of Th17 cell attenuation during CIA.

To confirm At’s indirect effects on Th17 cells, lymph node cells were isolated from immunized mice and tested for expression of *IL-2*, *IL-17*, *IL-22*, and *FasL*. Interestingly, only *IL-17* expression was significantly reduced in mice treated with At after CIA development, although all four genes were downregulated when mice were given At from the first collagen immunization. These gene expression patterns reflect our histological findings indicating a lower efficacy of At with respect to cartilage protection when administered after appearance of CIA signs and symptoms.

Additional evidence of At’s inhibitory effects on Th17 cell development relates to their polarization from splenic CD4^+^ T cells, which was blocked by At, even in the presence of exogenous IL-6/TGF-β and anti-IL-4 and -IL-12 mAbs. Decreased IL-17 and RORC, a Th17-specific transcription factor, further confirmed At’s modulation of Th17 cell activity. Notably, anti-IL-17 mAbs have been used unsuccessfully to treat RA in human patients (McInnes and Schett, 2017), which seemingly conflicts with our findings. We propose that inhibition of IL-17 alone is not sufficient to fully reverse RA, as multiple Th17-associated cytokines are involved in RA pathogenesis and are modulated by At. Alternatively, this disparity could be due to physiological differences between RA in humans and mice. However, we cannot exclude that inhibition of Th17 function is not the main therapeutic mechanism of At’s decoction; regardless, the potential use of IL-17 mAbs in conjunction with inhibitors of other Th17-specific mediators to treat RA warrants further study.

Finally, we determined that a dominant component of At leaf decoction is TAT-2, a flavonoid glucoside, that contributed to decreased differentiation of Th17 cells based on measured transcription levels of *IL-17* and *RORC*. The effect of TAT-2 alone on the expression of these genes was not statistically significant, suggesting that synergistic effects of multiple At decoction compounds may be necessary to produce significant effects. Because of the essentially negative *in vitro* results for TAT-2, performing additional experiments to examine the *in vivo* clinical efficacy of TAT-2 in a CIA model was not warranted. Our future efforts will focus on isolating and testing At’s other major components, such as TAT-6, for their potential inhibitory effects on Th17 cell differentiation and/or function, both alone and in combination with TAT-2. The potential effects of artonkin-4′-*O*-glucoside, another substance isolated from *A. tonkinensis* leaves, which has been shown to suppress experimentally induced arthritis in a rat model of CIA (Dang et al., 2009), may also be explored alone or in combination with TAT-2.

In conclusion, although some questions remain regarding the mechanism of At-mediated Th17 cell attenuation, our findings highlight the importance of investigating traditional medicines for treatment of diseases. The potential anti-inflammatory activity of At provides an alternative RA therapy apart from methotrexate and corticosteroids that should be explored further with respect to its optimal efficacy and possible non-specific cytotoxic effects, since comprehensive studies regarding At decoction toxicity have not yet been performed.

## Ethics Statement

The study has been approved by the ethical commission of the University of Perugia and the Italian Ministry of Health.

## Author Contributions

SA and IM performed the experiments and conducted the research. AF contributed to the autoimmune knowledge. AL and CR contributed to the spleen and pharmacology knowledge and correcting the manuscript. MC performed the histology assessment. LC and FB performed the HPLC experiments. TQ, NT, TS, and TT harvested, stored, dried plant leaves, and isolated their main compound. DD led the research.

## Conflict of Interest Statement

The authors declare that the research was conducted in the absence of any commercial or financial relationships that could be construed as a potential conflict of interest.

## Abbreviations

At: *Artocarpus tonkinensis* decoction
IL: interleukin; CIA, collagen-induced arthritis
mAbs: monoclonal antibodies
RA: rheumatoid arthritis
RANKL: receptor activator of nuclear factor kappa-B ligand
VTM: Vietnamese traditional medicine.

## References

[B1] Adorisio S. Fierabracci A. Rossetto A. Muscari I. Nardicchi V. Liberati A. M. (2016). Integration of traditional and western medicine in vietnamese populations: a review of health perceptions and therapies. *Nat. Prod. Commun.* 11 1409–1416. 30807048

[B2] Blasi F. Urbani E. Simonetti M. S. Chiesi C. Cossignani L. (2016). Seasonal variations in antioxidant compounds of Olea europaea leaves collected from different Italian cultivars. *J. Appl. Bot. Food Qual.* 89 202–207. 10.5073/Jabfq.2016.089.025

[B3] Brand D. D. Latham K. A. Rosloniec E. F. (2007). Collagen-induced arthritis. *Nat. Protoc.* 2 1269–1275. 10.1038/nprot.2007.173 17546023

[B4] Colin E. M. Asmawidjaja P. S. van Hamburg J. P. Mus A. M. van Driel M. Hazes J. M. (2010). 1,25-dihydroxyvitamin D3 modulates Th17 polarization and interleukin-22 expression by memory T cells from patients with early rheumatoid arthritis. *Arthritis Rheum.* 62 132–142. 10.1002/art.25043 20039421

[B5] Dang D. T. Eriste E. Liepinsh E. Trinh T. T. Erlandsson-Harris H. Sillard R. (2009). A novel anti-inflammatory compound, artonkin-4’-O-glucoside, from the leaves of Artocarpus tonkinensis suppresses experimentally induced arthritis. *Scand. J. Immunol.* 69 110–118. 10.1111/j.1365-3083.2008.02205.x 19170963

[B6] Delfino D. V. Pozzesi N. Pierangeli S. Ayroldi E. Fierabracci A. (2011). Manipulating thymic apoptosis for future therapy of autoimmune diseases. *Curr. Pharm. Des.* 17 3108–3119. 21864269 10.2174/138161211798157621

[B7] Dung T. N. Bodeker G. (2001). Tue Tinh: founder of vietnamese traditional medicine. *J. Altern. Complement. Med.* 7 401–403. 10.1089/10755530152639710 11719941

[B8] Geboes L. Dumoutier L. Kelchtermans H. Schurgers E. Mitera T. Renauld J. C. (2009). Proinflammatory role of the Th17 cytokine interleukin-22 in collagen-induced arthritis in C57BL/6 mice. *Arthritis Rheum.* 60 390–395. 10.1002/art.24220 19180498

[B9] Horwood N. J. Kartsogiannis V. Quinn J. M. Romas E. Martin T. J. Gillespie M. T. (1999). Activated T lymphocytes support osteoclast formation in vitro. *Biochem. Biophys. Res. Commun.* 265 144–150. 10.1006/bbrc.1999.1623 10548505

[B10] Kaneko S. Kondo Y. Yokosawa M. Furuyama K. Segawa S. Tsuboi H. (2018). The RORγt-CCR6-CCL20 axis augments Th17 cells invasion into the synovia of rheumatoid arthritis patients. *Mod. Rheumatol.* 28 814–825. 10.1080/14397595.2017.1416923 29251019

[B11] Kong Y. Y. Feige U. Sarosi I. Bolon B. Tafuri A. Morony S. (1999). Activated T cells regulate bone loss and joint destruction in adjuvant arthritis through osteoprotegerin ligand. *Nature* 402 304–309. 10.1038/46303 10580503

[B12] Kotake S. Udagawa N. Takahashi N. Matsuzaki K. Itoh K. Ishiyama S. (1999). IL-17 in synovial fluids from patients with rheumatoid arthritis is a potent stimulator of osteoclastogenesis. *J. Clin. Invest.* 103 1345–1352. 10.1172/JCI5703 10225978 PMC408356

[B13] Kuwabara T. Ishikawa F. Kondo M. Kakiuchi T. (2017). The role of IL-17 and related cytokines in inflammatory autoimmune diseases. *Mediators Inflamm.* 2017:3908061. 10.1155/2017/3908061 28316374 PMC5337858

[B14] Liu Q. X. Dai M. (2012). Medical exchanges between china and vietnam during the song, jin and yuan periods. *Zhonghua Yi Shi Za Zhi* 42 18–20. 22613476

[B15] Lu S. Wang Q. Li G. Sun S. Guo Y. Kuang H. (2015). The treatment of rheumatoid arthritis using Chinese medicinal plants: from pharmacology to potential molecular mechanisms. *J. Ethnopharmacol.* 176 177–206. 10.1016/j.jep.2015.10.010 26471289

[B16] McInnes I. B. Schett G. (2017). Pathogenetic insights from the treatment of rheumatoid arthritis. *Lancet* 389 2328–2337. 10.1016/S0140-6736(17)31472-1 28612747

[B17] Nakae S. Saijo S. Horai R. Sudo K. Mori S. Iwakura Y. (2003). IL-17 production from activated T cells is required for the spontaneous development of destructive arthritis in mice deficient in IL-1 receptor antagonist. *Proc. Natl. Acad. Sci. U.S.A.* 100 5986–5990. 10.1073/pnas.1035999100 12721360 PMC156313

[B18] Ngoc D. D. Catrina A. I. Lundberg K. Harris H. E. Ha N. T. Anh P. T. (2005). Inhibition by artocarpus tonkinensis of the development of collagen-induced arthritis in rats. *Scand. J. Immunol.* 61 234–241. 10.1111/j.1365-3083.2005.01560.x 15787740

[B19] Ogura H. Murakami M. Okuyama Y. Tsuruoka M. Kitabayashi C. Kanamoto M. (2008). Interleukin-17 promotes autoimmunity by triggering a positive-feedback loop via interleukin-6 induction. *Immunity* 29 628–636. 10.1016/j.immuni.2008.07.018 18848474

[B20] Pelaia G. Vatrella A. Maselli R. (2012). The potential of biologics for the treatment of asthma. *Nat. Rev. Drug Discov.* 11 958–972. 10.1038/nrd3792 23197041

[B21] Pozzesi N. Gizzi S. Gori F. Vacca C. Cannarile L. Riccardi C. (2007). IL-2 induces and altered CD4/CD8 ratio of splenic T lymphocytes from transgenic mice overexpressing the glucocorticoid-induced protein GILZ. *J. Chemother.* 19 562–569. 10.1179/joc.2007.19.5.562 18073156

[B22] Pozzesi N. Pierangeli S. Vacca C. Falchi L. Pettorossi V. Martelli M. P. (2011). Maesopsin 4-O-beta-D-glucoside, a natural compound isolated from the leaves of Artocarpus tonkinensis, inhibits proliferation and up-regulates HMOX1, SRXN1 and BCAS3 in acute myeloid leukemia. *J. Chemother.* 23 150–157. 10.1179/joc.2011.23.3.150 21742584

[B23] Sarkar S. Tesmer L. A. Hindnavis V. Endres J. L. Fox D. A. (2007). Interleukin-17 as a molecular target in immune-mediated arthritis: immunoregulatory properties of genetically modified murine dendritic cells that secrete interleukin-4. *Arthritis Rheum.* 56 89–100. 10.1002/art.22311 17195211

[B24] Sato K. Suematsu A. Okamoto K. Yamaguchi A. Morishita Y. Kadono Y. (2006). Th17 functions as an osteoclastogenic helper T cell subset that links T cell activation and bone destruction. *J. Exp. Med.* 203 2673–2682. 10.1084/jem.20061775 17088434 PMC2118166

[B25] Thuy T. T. Kamperdick C. Ninh P. T. Lien T. P. Thao T. T. Sung T. V. (2004). Immunosuppressive auronol glycosides from Artocarpus tonkinensis. *Pharmazie* 59 297–300. 15125577 10.1002/chin.200431190

[B26] Thuy T. T. Thien D. D. Hung T. Q. Tam N. T. Anh N. T. H. Dung L. K. (2017). Flavonol and proanthocyanidin glycosides from the leaves of artocarpus tonkinensis. *Chem. Nat. Compd.* 53 759–761. 10.1007/s10600-017-2113-1

[B27] Thuy T. T. Thien D. D. Quang Hung T. Tam N. T. Anh N. T. Nga N. T. (2016). In vivo anticancer activity of maesopsin 4-O-beta-glucoside isolated from leaves of Artocarpus tonkinensis A. Chev. Ex Gagnep. *Asian Pac. J. Trop. Med.* 9 351–356. 10.1016/j.apjtm.2016.03.012 27086153

