# Supplementary material for: Artocarpus tonkinensis Protects Mice Against Collagen-Induced Arthritis and Decreases Th17 Cell Function
Source: Front Pharmacol. 2019 May 31;10:503. doi: 10.3389/fphar.2019.00503 (PMC6554681; doi:10.3389/fphar.2019.00503)
Supplement: Supplementary file 1 [file Data_Sheet_1.docx]

**Material and Methods**

**Mice**: DBA1/J, females, 8-12 weeks old, supplied by Biogem scarl Animal House Ariano Irpino, AV, Italy were used. Mice have been housed inside cages of poliosulfone (3-5 mice/cage) with stainless cover-feed and sterilized and dust-free bedding cobs. Mice were maintained in cages with paper filter covers; food and bedding were sterilized. Animals have been housed under a light-dark cycle, keeping temperature and humidity constant. Parameters of the animal rooms have been assessed as follows: 22+2 ^0^C temperature, 55+105 relative humidity, about 15-20 filtered air changes/hour and 12 hour circadian cycle of artificial light (7 a.m., 7 p.m.).

Diet and water supply: Drinking water has been supplied ad libitum. Each mouse has been offered daily a complete pellet diet (GLP $RF21, Mucedola) throughout the study. The analytical certificates of animal food and water have been retained at Biogem premises.

Experimental Design:

**CIA**: The collagen-induced arthritis (CIA) has been elicited by immunization with CII (Collagen from chicken stema, Sigma, C9301-5MG, Lot#016M4158V) emulsified in Complete Freund’s Adjuvant (CFA, F5881-10ML, Lot#SLBQ1106V) or Incomplete Freund’s Adjuvant (CFA, F5506-10ML, Lot#SLBL9742V) by intradermal injections (50μL/mouse). Arthritis has been induced on 15 mice and further 3 mice represented the negative untreated control group.

The following describes the different phases of the procedure:

*Preparation of emulsion:*

For the emulsion have been calculated the volumes of collagen and CFA (Day +1) or IFA (Day +31) required making the emulsion, keeping their ratios at 1:1. In order to inject 50μL of emulsion per mouse, extra emulsion has been made owing to both losses during processing and the dead space in the syringes. All reagents have been kept on ice. The emulsion has been mixed for 20 minutes. When the emulsion was ready, it has been transferred in a sterile eppendorf tube, then to a sterile 1 mL syringe for immunization by using a 18G needle. The 18G needle has been then replaced with 26 x ½ gauge needle, kept on ice, ready for intradermal (i.d.) mouse injection.

*Immunization:*

The injection has been performed at about 1,5 cm distal from the base of the tail, being careful to choose a tissue site and not a vessel. 50μL of emulsion have been slowly injected intradermally into the tail while the mice has been used under gas anesthesia (2% isoflurane).

*Monitoring of arthritis incidence:*

Animals have been evaluated for arthritis incidence as follows:

1. By clinical observations two times per week for 5 consecutive weeks;
2. By caliper measurement;
3. By IVIS bioluminescence acquisition.

**Identification of animals and allocation to groups**

Animals have been numbered by ear punch (from n^o^1 till n^o^18). The cages have been identified by a paper tag indicating: number of mice, group, CIA induction, date of induction, treatment.

**Artocarpus tonkinensis treatment**

Mice have been divided in 4 groups of 3/5 animals each and treated as described below:

| **Gp** | **N^o^** | **CIA** | **CIA schedule**  **(CFA or IFA)** | **Treatment** | **Schedule** | **Sacrifice** |
| --- | --- | --- | --- | --- | --- | --- |
| 1 | 5 | 50μL/mouse | Day +1* and Day +31 | Water | Day +1 | +48 |
| 2 | 5 | 50μL/mouse | Day +1* and Day +31 | *Artocarpus*  *tonkinensis* | Day +1 | +48 |
| 3 | 5 | 50μL/mouse | Day +1* and Day +31 | *Artocarpus*  *tonkinensis* | From CIA development | +49 |
| 4 | 3 | - | - | Water | Day +1 | +48 |

*Day +1 is considered as the first day of arthritis induction

**Type and frequency of recording**

**Mortality**

Animals have been inspected every day for mortality.

**Clinical observations**

Physical appearance, behavior and general and local clinical signs of the mice have been observed daily. Any deviation from normality has been observed.

To follow arthritis development, each paw has been evaluated and scored individually on a scale 0-4, with 4 indicating the most severe inflammation (table 1).

**Table 1.** Scoring system for subjective evaluation of arthritis severity

| **Severity score** | **Degree of inflammation** |
| --- | --- |
| ***0*** | No evidence of erythema and swelling |
| ***1*** | Erythema and mild swelling confined to the tarsals or ankle joint |
| ***2*** | Erythema and mild swelling extending from the ankle to the tarsals |
| ***3*** | Erythema and moderate swelling extending from the ankle to metatarsal joints |
| ***4*** | Erythema and severe swelling encompass the ankle, foot and digits, or ankylosis of the limb |

**Body weight**

All animals have been weighed during the experimental period. The body weight loss % (BWL%) was determined as follows: BWL% = 100 – (mean BW_day x_/mean BW_day 1_ x 100), where BW_x_ is the mean BW at the examined day and BW_1_ is the mean on the 1^st^ day of experimental period.

**Test Item consumption**

Artocarpus tonkinensis consumptions has been evaluated weekly.

**Caliper measurements**

Weekly the thickness of the joints has been evaluated by Milutoyo caliper measurement.

**IVIS chemiluminescence acquisitions**

Arthritis onset has been followed with IVIS Spectrum (PerlinElmer) chemiluminescence acquisition, by injecting Lucigenin 25mg/kg, i.p., [Sigma-Aldrich, #M8010]. Bioluminescence imaging has been performed 10 minutes after intraperitoneal Lucigenin (25mg/kg) injection using the IVIS Spectrum (PerkinElmer), to evaluate the inflammatory degree at joint level.

**Figure 1**

Figure 1. IVIS acquisition at different time points (day +14 and day +20) of animals enrolled in the experimental groups. Images were acquired with IVIS Spectrum (PerkinElmer) 10 minutes after Lucigenin intraperitoneal injection (25 mg/kg). For each acquisition were used the following parameters: Exposition: 1 minute; Binning: 1; F/Stop: 1.

**Sacrifice**

Animals were sacrificed by CO_2_ asphyxia.

**Isolation of CD4^+^ T lymphocytes from mice spleen.**


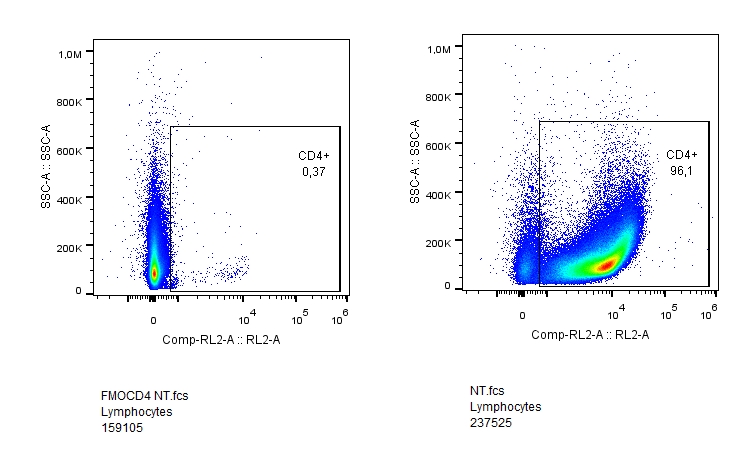

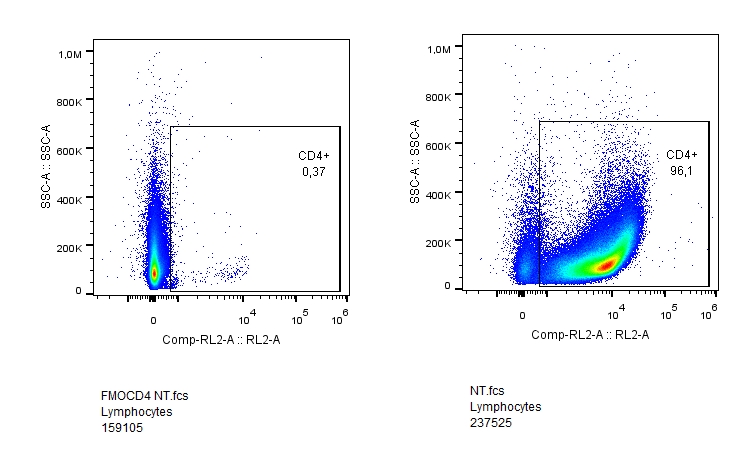


Figure 2: Flow cytometry analysis shows a representative experiment in which the purity of the isolated CD4^+^ T lymphocytes from the spleen of C57BL/6 mice was > 95%.

**Consumption of At decoction compared to water in CIA or healthy mice**

Figure 3: Amount of *A. tonkinensis* decoction (mL/mouse, *y*-axis) consumed by mice during the course of experiment (weeks, *x-*axis). The consumption of At decoction (gray line, CIA+AT (from day 1) was higher compared to consumption of water in immunized (CIA) or healthy (Control) mice.
